# Optimization of antipsychotic use in the elderly with severe mental illness; a review of three cases.

**DOI:** 10.1192/j.eurpsy.2023.1972

**Published:** 2023-07-19

**Authors:** A. Izquierdo De La Puente, I. Ramos García, G. Bracchitta, E. Martín Ferrer

**Affiliations:** 1 Psychiatry; 2 Medicine; 3 neuropsychology, ORPEA Puerta de Hierro, Madrid, Spain

## Abstract

**Introduction:**

A descriptive study is presented on three cases in which the adjustment of antipsychotic treatment has led to an improvement in the patients’ quality of life.

**Objectives:**

The objective is the description of the process of treatment optimization in elderly people, with the secondary improvement in the quality of life.

**Methods:**

These are three female patients, with an average age of 73 years, institutionalized in the ORPEA Puerta de Hierro Specialized Mental Health Center for the Elderly. Two of them have a diagnosis of paranoid schizophrenia, and the third has a diagnosis of delusional disorder. The average age at the onset of symptoms was 21 years old.

All three were receiving treatment with biweekly zuclopenthixol, 200mg DMD together with haloperidol at a mean dose of 7.5mg of haloperidol. For the extrapyramidal side effects presented they were on treatment with biperiden 4mg DMD.

In addition to psychopathological examinations and subjective impressions, previous and current status were compared with the Barthel Scale, GDS and family satisfaction scale.

**Results:**

The treatment of the three patients was modified, in a period of three months, replacing the treatment with zuclopenthixol 200mg DMD to paliperidone extended release 150mg every 21 days, as well as with oral paliperidone, at an average dose of 10mg DMD. Also, biperiden treatment could be completely withdrawn and the dose of haloperidol could be reduced to 2.25mg.

In addition to the reduction of polypharmacy doses, an overall improvement is observed in relation to psychotic symptoms, without presenting exacerbations and improvement in the control of chronic psychotic symptoms. Likewise, they have improved at the affective level, presenting less negative symptoms, improvement of the affective flattening, being more resonant in the interaction with peers and with increased participation in joint activities. On the other hand, at a cognitive level, a significant improvement has been observed.

**Conclusions:**

First generation antipsychotics produce a high rate of side effects, such as prolactin increase, affective blunting and extrapyramidal symptoms. In contrast, second-generation antipsychotics, in addition, act as 5HT2A antagonists, thus reducing the rates of unwanted effects.

Some studies have shown that paliperidone increases prolactin to a lesser extent than risperidone and causes fewer extrapyramidal and cognitive effects. Furthermore, because of its safety profile, paliperiona may be a first-choice strategy in psychosis in the elderly, even in its intramuscular extended-release form in nonadherent patients.

In conclusion, the elderly are a vulnerable population and to a greater extent when they suffer from a severe mental disorder. The aim of any treatment must be directed not only to control positive symptoms, but also to slow down the deterioration of the disease itself, as well as to improve quality of life and functionality.

**Disclosure of Interest:**

None Declared

